# Molecular Simplification of Natural Products: Synthesis, Antibacterial Activity, and Molecular Docking Studies of Berberine Open Models

**DOI:** 10.3390/biomedicines9050452

**Published:** 2021-04-22

**Authors:** Gualtiero Milani, Maria Maddalena Cavalluzzi, Roberta Solidoro, Lara Salvagno, Laura Quintieri, Angela Di Somma, Antonio Rosato, Filomena Corbo, Carlo Franchini, Angela Duilio, Leonardo Caputo, Solomon Habtemariam, Giovanni Lentini

**Affiliations:** 1 Department of Pharmacy–Pharmaceutical Sciences, University of Bari Aldo Moro, via E. Orabona n. 4, 70126 Bari, Italy; gualtiero.milani@uniba.it (G.M.); roberta.solidoro@uniba.it (R.S.); l.salvagno@studenti.uniba.it (L.S.); antonio.rosato@uniba.it (A.R.); filomena.corbo@uniba.it (F.C.); carlo.franchini@uniba.it (C.F.); giovanni.lentini@uniba.it (G.L.); 2 Institute of Sciences of Food Production (CNR-ISPA) National Council of Research, Via G. Amendola, 122/O, 70126 Bari, Italy; laura.quintieri@ispa.cnr.it (L.Q.); leonardo.caputo@ispa.cnr.it (L.C.); 3 Department of Chemical Sciences, University of Naples “Federico II” Via Cinthia 4, 80126 Napoli, Italy; angela.disomma@unina.it (A.D.S.); angela.duilio@unina.it (A.D.); 4 Pharmacognosy Research Laboratories & Herbal Analysis Services, University of Greenwich, Chatham-Maritime, Kent ME4 4TB, UK; s.habtemariam@herbalanalysis.co.uk

**Keywords:** berberine, molecular simplification, antibacterial activity, FtsZ, ‘fragment’, ligand efficiency

## Abstract

Berberine, the main bioactive component of many medicinal plants belonging to various genera such as *Berberis*, *Coptis*, and *Hydrastis* is a multifunctional compound. Among the numerous interesting biological properties of berberine is broad antimicrobial activity including a range of Gram-positive and Gram-negative bacteria. With the aim of identifying berberine analogues possibly endowed with higher lead-likeness and easier synthetic access, the molecular simplification approach was applied to the secondary metabolite and a series of analogues were prepared and screened for their antimicrobial activity against Gram-positive and Gram-negative bacterial test species. Rewardingly, the berberine simplified analogues displayed 2–20-fold higher potency with respect to berberine. Since our berberine simplified analogues may be easily synthesized and are characterized by lower molecular weight than the parent compound, they are further functionalizable and should be more suitable for oral administration. Molecular docking simulations suggested FtsZ, a well-known protein involved in bacterial cell division, as a possible target.

## 1. Introduction

Since antiquity, medicinal plants have been serving as the largest biochemical and pharmaceutical living stores for treating human diseases. Endless series of biologically relevant compounds [1] including numerous natural antimicrobial agents [2,3] have been sourced from medicinal plants. A vast number of medicinal plants for treating infectious diseases such as urinary tract infections, gastrointestinal disorders, respiratory diseases, and cutaneous infections have also been recognized [4]. Though the discovery of antibiotics nearly a century ago has revolutionized antibacterial chemotherapy, their widespread and sometimes irrational use have resulted in the ever-increasing appearance of antibiotics-resistant strains. Furthermore, no new class of antibiotics has been discovered for a long time and the antibiotics that entered the markets in recent years were analogues of existing molecules [5]. The continued nemesis of new resistant pathogenic strains on world health means that we should look for new and more potent therapeutics to fight deadly microbial infections [6]. In this connection, new strategies that accelerate the discovery and development of innovative antimicrobial agents must be investigated. Medicinal plants represent a paramount area of research, given the wide availability of phytochemicals with structural and functional diversity [3] that could become, with appropriate modifications, a clinically relevant therapeutic source. Furthermore, in terms of drug-like properties, phytochemicals benefit from biological prevalidation: their core scaffolds can be considered naturally privileged structures in drug discovery since they have already interacted with different enzymes and proteins during their biosynthesis. Therefore, they inherently fall into the biologically relevant chemical space, seeming predestined for interaction with drug targets [7,8,9]. Unfortunately, natural products show some drawbacks as their isolation is difficult and time-consuming and their total synthesis is not so amenable to large-scale production, due to their high structural complexity and relatively large molecular weight. These features, moreover, can convey unfavorable absorption, distribution, metabolism, excretion, and toxicity (ADMET) profiles. In some cases, standard structure–activity relationship (SAR) approaches based on structural modifications to natural products fail to generate drug-like derivatives and, rather, generate compounds still presenting the so-called molecular obesity [10]. Since redundant atoms do not participate in the binding to the target, it should be advisable to properly remove them drawing synthetically more accessible simplified analogues. This widely used powerful strategy, known as structural simplification, often improves the success rate of natural product-based drug development, leading to the chemical synthesis of smaller fragments retaining, or sometimes improving, desired biological parameters such as potency and/or selectivity [11,12].

**Figure 1:**
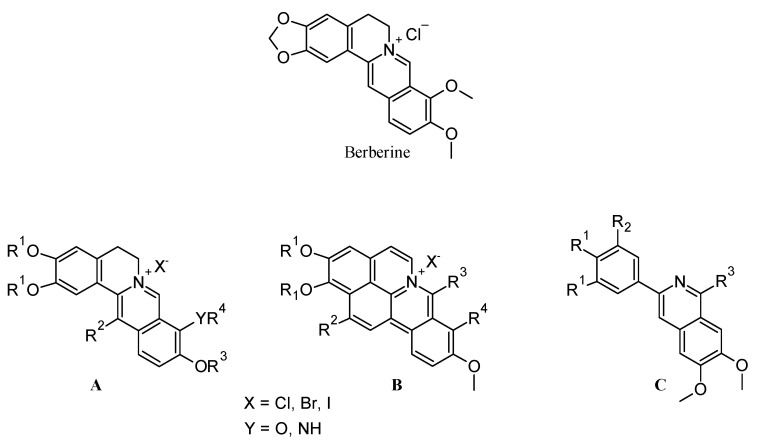
General structures of most of the antimicrobial berberine derivatives reported in the literature (**A**–**C**).

Many plant species widely explored for their antimicrobial potential belong to the families of *Berberidaceae, Ranunculaceae, Rutaceae,* and *Annonaceae* [13,14,15]. Within these families, the genus *Berberis* is well documented for its antimicrobial properties, besides other biological activities such as antihypertensive, anti-inflammatory, antioxidant, antidepressant, anticancer, antidiarrheal, antidiabetic, and hepatoprotective [15]. In particular, berberine—the main secondary metabolite of *Berberis*—is an antimicrobial agent acting against a wide variety of microorganisms including Gram-positive and Gram-negative bacteria, fungi, protozoa, trypanosomes, and plasmodia [16]. To the best of our knowledge, very few researchers applied the structural simplification approach to obtain ‘slimmer’ analogues of berberine endowed with antibacterial properties. While many antimicrobial berberine derivatives have already been reported in the literature (structures **A** and **B**, Figure 1) [17,18,19,20,21,22,23,24,25,26,27,28,29], only one article has been dedicated to antimicrobial berberine simplified analogues based on a 3-phenyl substituted 6,7-dimethoxyisoquinoline scaffold (structure **C**, Figure 1) [30]. Concerning the latter assemblage **C**, albeit showing a less complex structure than berberine, the related compounds are still mostly rigid and flat due to the two aromatic ring systems directly linked together.

**Figure 2:**
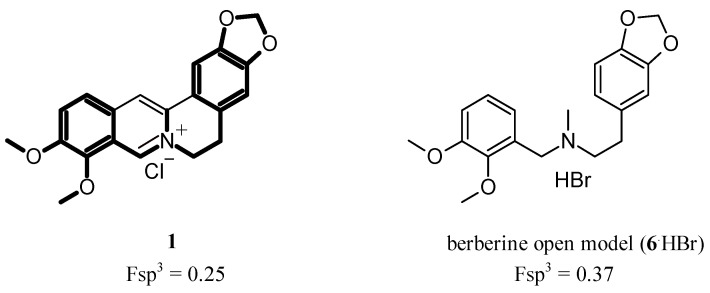
Structures of both berberine (**1**) and its open model (**6**^.^HBr).

Our first goal was to prepare more flexible open models of berberine endowed with a higher Fsp^3^ fraction (Figure 2), a parameter known to enhance the bioavailability and selectivity of compounds, thus making them more attractive for the drug discovery process [31]. Therefore, starting from a series of berberine simplified analogues previously synthesized in our laboratory [32], novel analogues have been prepared and their antibacterial activity evaluated. Since it has been reported that berberine can inhibit the filamentous temperature-sensitive Z protein (FtsZ) [21,33,34], a well-known bacterial protein involved in cell division, a 3D quantitative structure–activity relationship (3D-QSAR) study on this target has also been performed.

## 2. Experimental Section

### 2.1. Chemistry

All chemicals were purchased from Sigma–Aldrich or Lancaster at the highest quality commercially available. Solvents were RP grade unless otherwise indicated. Yields refer to purified products and were not optimized. The structures of the compounds were confirmed by routine spectrometric and spectroscopic analyses. Only spectra for compounds not previously described are given, and only unambiguous ^13^C signals were assigned. Compounds **5a**, **5b**, **10**, and **13**–**15** were obtained as previously reported [32]. Melting points were determined on a Gallenkamp apparatus in open glass capillary tubes and are uncorrected. ^1^H and ^13^C NMR spectra were recorded on a Varian Mercury-VX spectrometer, operating at 300 and 75 MHz for ^1^H and ^13^C, respectively, or on an Agilent 500-vnmrs500 spectrometer, operating at 500 and 125 MHz for ^1^H and ^13^C, respectively, using CDCl_3_ as solvent, unless otherwise indicated. Chemical shifts are reported in parts per million (ppm) relative to solvent resonance: CDCl_3_, δ 7.26 (^1^H NMR) and δ 77.3 (^13^C NMR). *J* values are given in Hz. EIMS spectra were recorded on a Hewlett-Packard 6890–5973 MSD gas chromatograph/mass spectrometer (Hewlett-Packard, Palo Alto, CA, USA) at low resolution. ESI^+^/^–^/MS/MS analyses were performed with an Agilent 1100 series LC–MSD trap system VL Workstation (Agilent, Palo Alto, CA, USA). Elemental analyses were performed with a Eurovector Euro EA 3000 analyzer. Chromatographic separations were performed on silica gel columns by flash chromatography (Kieselgel 60, 0.040–0.063 mm, Merck, Darmstadt, Germany). TLC analyses were performed on precoated silica gel on aluminum sheets (Kieselgel 60 F254, Merck).

#### 2.1.1. 2-(3,4-Methylenedioxyphenyl)ethylamine (3)

Prepared as reported in the literature [32]. Yield: 85%, oil; MS (70 eV) *m*/*z* (%) 165 (M^+^, 17), 136 (100). Spectroscopic data were in agreement with those reported in the literature [32].

#### 2.1.2. General Procedure for the Synthesis of Compounds **5**c–e

2-(*H*-1,3-Benzodioxol-5-yl)-*N*-[(4-chlorophenyl)methyl]ethan-1-amine hydrochloride (**5c**^.^HCl)

The procedure adopted for the synthesis of 2-(2*H*-1,3-benzodioxol-5-yl)-*N*-[(4-chlorophenyl)methyl]ethan-1-amine hydrochloride (**5c**^.^HCl) is described.

A solution of 2-(3,4-methylenedioxyphenyl)ethylamine (**3**) (0.33 g, 2.0 mmol) and 4-chlorobenzaldehyde (0.28 g, 2.0 mmol) in 5 mL of dry CH_2_Cl_2_ was stirred at room temperature overnight in the presence of 3 Å molecular sieves. The mixture was then filtered through celite and CH_2_Cl_2_ was removed under vacuum to obtain 0.59 g of a yellowish oil (**4c**) which was reacted without further purification. To a stirred solution of imine **4c** in MeOH (15 mL) and CH_2_Cl_2_ (5 drops), sodium borohydride (0.117 g, 3.1 mmol) was added and the reaction mixture was heated at reflux for 3 h. After the mixture was cooled to 0 °C, water was added, and methanol was removed under vacuum. The aqueous phase was extracted three times with CH_2_Cl_2_ and the combined organic phases were dried over anhydrous Na_2_SO_4_. The solvent was removed under vacuum to give 0.495 g (84%) of **5c** as a yellowish oil: GC-MS (70 eV) *m*/*z* (%): 289 (M^+^ < 1), 125 (100). The corresponding hydrochloride (**5c**^.^HCl) was obtained dissolving the free base in 1 mL of 2 M HCl and azeotropically removing water (toluene/abs EtOH). The obtained white solid was recrystallized from MeOH/Et_2_O giving 0.28 g (42%) of white crystals: mp > 250 °C; ^1^H NMR (500 MHz, DMSO-*d*_6_): *δ* 2.92 (dd, *J* = 9.7, 6.4 Hz, 2H, C*H*_2_CH_2_N), 3.05 (dd, *J* = 9.9, 6.4 Hz, 2H, NC*H*_2_CH_2_), 4.14 (s, 2H, C*H*_2_N), 5.98 (s, 2H, OC*H*_2_O), 6.70 (dd, *J* = 7.9, 1.7 Hz, 1H, benzodioxole *H*C-6), 6.84 (d, *J =* 1.6 Hz, 1H, benzodioxole *H*C-4), 6.85 (d, *J =* 8.0 Hz, 1H, benzodioxole *H*C-7), 7.50 (d, *J* = 8.5 Hz, 2H, benzyl *H*C-2,6), 7.61 (d, *J* = 8.5 Hz, 2H, benzyl *H*C-3,5), 9.55 (br s, 2H, NH_2_^+^); ^13^C NMR (125 MHz, DMSO-*d*_6_): *δ* 31.1 (1C, *C*H_2_), 47.7 (1C, *C*H_2_), 49.0 (1C, *C*H_2_), 100.9 (1C, O*C*H_2_O), 108.4 (1C, Ar-*C*H), 109.0 (1C, Ar-*C*H), 121.7 (1C, Ar-*C*H), 128.6 (2C, phenyl *C*-2,6 or *C*-3,5), 130.9 (1C, *C*q), 131.1 (1C, *C*q), 132.1 (2C, phenyl *C*-3,5 or *C*-2,6), 133.7 (1C, *C*q), 146.0 (1C, benzodioxole *C*-7a or 3a), 147.4 (1C, benzodioxole *C*-3a or 7a); HRMS (QTOF, *m*/*z*) calcd for C_16_H_16_ClNO_2_: 290.0942 ([M + H]^+^); found 290.0947; Anal. Calcd for C_16_H_16_ClNO_2_^.^0.5HCl^.^0.5MeOH: C, 61.16; H, 5.76; N, 4.32; found: C, 61.38; H, 5.62; N, 4.36.

2-(*H*-1,3-Benzodioxol-5-yl)-*N*-[(2-nitrophenyl)methyl]ethan-1-amine hydrochloride (**5d**^.^HCl)

Prepared as described above for **5c** starting from **3** and 2-nitrobenzaldheyde in 96% yield. GC-MS (70 eV) *m*/*z* (%): 300 (M^+^, <1), 136 (100).

Data for **5d**^.^HCl (yellow crystals, 36%): mp 234–236 °C; ^1^H NMR (500 MHz, CD_3_OD + D_2_O): *δ* 3.02 (t, *J* = 7.8 Hz, 2H, C*H*_2_CH_2_N), 3.43 (t, *J* = 7.8 Hz, 2H, NC*H*_2_CH_2_), 4.50 (s, 2H, C*H*_2_N), 5.95 (s, 2H, OC*H*_2_O), 6.78–6.88 (m, 3H, benzodioxole *H*C-4,6,7), 7.70 (d, *J =* 7.6 Hz, 1H, benzyl *H*C-6), 7.76 (apparent t, 1H, benzyl *H*C-4), 7.85 (apparent t, 1H, benzyl *H*C-5), 8.28 (d, *J* = 8.2 Hz, 1H, benzyl *H*C-3); ^13^C NMR (125 MHz, CD_3_OD + D_2_O): *δ* 32.3 (1C, *C*H_2_), 49.5 (1C, *C*H_2_), 50.0 (1C, *C*H_2_), 102.2 (1C, O*C*H_2_O), 109.7 (1C, Ar-*C*H), 110.0 (1C, Ar-*C*H), 123.1 (1C, Ar-*C*H), 126.9 (1C, phenyl *C*-1), 127.1 (1C, Ar-*C*H), 131.0 (1C, Ar-*C*H), 132.6 (1C, Ar-*C*H), 134.9 (1C, *C*q), 136.2 (1C, Ar-*C*H), 147.5 (1C, benzodioxole *C*-7a or 3a), 148.7 (1C, benzodioxole *C*-3a or 7a), 149.4 (1C, phenyl *C*-2); HRMS (QTOF, *m*/*z*) calcd for C_16_H_16_N_2_O_4_: 323.1002 ([M + Na]^+^); found 323.1006; Anal. Calcd for C_16_H_16_N_2_O_4_^.^HCl^.^0.25 MeOH: C, 56.61; H, 5.26; N, 8.12; found: C, 56.92; H, 5.27; N, 7.86.

2-(*H*-1,3-Benzodioxol-5-yl)-*N*-[(naphthalen-1-yl)methyl]ethan-1-amine hydrochloride (**5e**^.^HCl)

Prepared as described above for **5c** starting from **3** and naphthalene-1-carbaldehyde in 86% yield. GC-MS (70 eV) m/z (%): 305 (M^+^, <1), 141 (100).

Data for **5e**^.^HCl (white crystals, 31%): mp 181–183 °C; ^1^H NMR (500 MHz, DMSO-*d*_6_): *δ* 2.98 (dd, *J* = 9.7, 6.5 Hz, 2H, C*H*_2_CH_2_N), 3.25 (dd, *J* = 9.8, 6.9 Hz, 2H, NC*H*_2_CH_2_), 4.65 (s, 2H, C*H*_2_N), 5.99 (s, 2H, OC*H*_2_O), 6.72 (dd, *J* = 8.0, 1.7 Hz, 1H, benzodioxole *H*C-6), 6.86 (d, *J =* 1.6 Hz, 1H, benzodioxole *H*C-4), 6.87 (d, *J =* 7.8 Hz, 1H, benzodioxole *H*C-7), 7.55–7.68 (m, 3H, naphth *H*C-2,3,6), 7.82 (apparent d, 1H, naphth *H*C-7), 8.01 (apparent d, 2H, naphth *H*C-4,5), 8.26 (d, *J* = 8.4 Hz, 1H, naphth *H*C-8), 9.51 (s, 2H, NH_2_^+^); ^13^C NMR (125 MHz, DMSO-*d*_6_): *δ* 31.4 (1C, *C*H_2_), 46.7 (1C, *C*H_2_), 48.5 (1C, *C*H_2_), 100.9 (1C, O*C*H_2_O), 108.4 (1C, Ar-*C*H), 109.0 (1C, Ar-*C*H), 121.7 (1C, Ar-*C*H), 123.8 (1C, Ar-*C*H), 125.4 (1C, Ar-*C*H), 126.3 (1C, Ar-*C*H), 126.8 (1C, Ar-*C*H), 128.3 (1C, Ar-*C*H), 128.7 (1C, Ar-*C*H), 129.1 (1C, Ar-*C*H), 129.6 (1C, *C*q), 131.0 (1C, *C*q), 131.1 (1C, *C*q), 133.3 (1C, *C*q), 146.0 (1C, benzodioxole *C*-7a or 3a),147.4 (1C, benzodioxole *C*-3a or 7a); HRMS (QTOF, *m*/*z*) calcd for C_20_H_19_NO_2_: 306.1489 ([M + H]^+^); found 306.1495; Anal. Calcd for C_20_H_19_NO_2_^.^HCl^.^0.1 MeOH: C, 69.97; H, 5.96; N, 4.06; found: C, 70.16; H, 6.46; N, 4.09.

#### 2.1.3. 2-(1,3-Benzodioxol-5-yl)-*N*-(2,3-dimethoxybenzyl)-*N*-methylethanamine hydrobromide (**6**^.^HBr)

A solution of iodomethane (0.02 mL, 0.30 mmol) in 1 mL of absolute EtOH was added dropwise to a magnetically stirred solution of **5a** (0.329 g, 1.05 mmol) and K_2_CO_3_ (0.290 g, 2.10 mmol) in abs EtOH (10 mL). The reaction mixture was stirred at room temperature for 24 h and then evaporated under reduced pressure. The residue was taken up with EtOAc and washed with water and brine. After drying (Na_2_SO_4_), the organic phase was evaporated under vacuum to afford 0.251 g (73%) of the desired compound (**6**) as a crude oil: GC-MS (70 eV) *m*/*z* (%): 329 (M^+^ < 1), 151 (100). The corresponding hydrobromide (**6**^.^HBr) was obtained dissolving the free base in 1 mL of 2 M HBr and azeotropically removing water (toluene/abs EtOH). The oil obtained was crystallized from abs EtOH/*i*Pr_2_O/hexane giving 0.096 g (25%) of white crystals: mp 128–129 °C; ^1^H NMR (500 MHz, DMSO-*d*_6_): δ 2.70 (d, *J =* 4.9 Hz, 3H, C*H*_3_N), 2.96 (t, *J* = 8.6 Hz, 2H, C*H*_2_CH_2_N), 3.16–3.24 (m, 1H, NC*H*HCH_2_), 3.26–3.32 (m, 1H, NCH*H*CH_2_), 3.81 (s, 3H, C*H*_3_O), 3.83 (s, 3H, C*H*_3_O), 4.22 (dd, *J* = 13.0, 6.6 Hz, 1H, C*H*HN), 4.43 (dd, *J* = 13.2, 3.9 Hz, 1H, CH*H*N), 5.98 (s, 2H, OC*H*_2_O), 6.72 (dd, *J* = 8.1, 1.7 Hz, 1H, phenyl *H*C-4), 6.860 (d overlapping s at 6.862, *J* = 7.3 Hz, 1H, benzodioxole *H*C-6), 6.862 (s overlapping d at 6.860, 1H, benzodioxole *H*C-4), 7.10 (dd, *J* = 6.9, 2.0 Hz, 1H, phenyl *H*C-6), 7.12–7.20 (m, 2H, phenyl *H*C-5 + benzodioxole *H*C-7), 9.37 (br s, 1H, NH^+^); ^13^C NMR (DMSO-*d*_6_, 500 MHz): δ 29.7 (1C, *C*H_2_), 53.6 (1C, *C*H_3_N), 56.3 (2C, *C*H_2_), 56.7 (1C, *C*H_3_O), 61.2 (1C, *C*H_3_O), 101.4 (1C, O*C*H_2_O), 108.9 (1C, Ar-*C*H), 109.6 (1C, Ar-*C*H), 115.2 (1C), 122.3 (1C, Ar-*C*H), 123.8 (1C, *C*q), 124.1 (1C, Ar-*C*H), 124.8 (1C, Ar-*C*H), 130.8 (1C), 146.5 (1C, benzodioxole *C*-7a or 3a), 147.9 (1C, benzodioxole *C*-3a or 7a), 148.2 (1C, phenyl *C*-2 or *C*-3), 152.8 (1C, phenyl *C*-3 or *C*-2). Anal. Calcd for C_19_H_23_NO_4_^.^HBr^.^0.5H_2_O: C, 54.42; H, 6.01; N, 3.34. found: C, 54.54; H, 5.77; N, 3.38.

#### 2.1.4. General Procedure for the Synthesis of Compounds **7**–**9**

2-(1,3-Benzodioxol-5-yl)-*N*-(2,3-dimethoxybenzyl)-*N*-ethylethanamine hydrobromide (**7**^.^HBr)

The procedure adopted for the synthesis of 2-(1,3-benzodioxol-5-yl)-*N*-(2,3-dimethoxybenzyl)-*N*-ethylethanamine (**7**^.^HBr) is described.

A solution of iodoethane (0.25 mL, 3.12 mmol) in 5 mL of absolute EtOH was added dropwise to a magnetically stirred solution of **5a** (0.82 g, 2.6 mmol) and K_2_CO_3_ (0.718 g, 5.2 mmol) in abs EtOH (20 mL). The reaction mixture was heated at 78 °C for 24 h and then evaporated under reduced pressure. The residue was taken up with EtOAc and washed with water and brine. After drying (Na_2_SO_4_), the organic phase was evaporated under vacuum to afford 0.812 g of a crude oil which was purified through column chromatography (EtOAc/hexane 3:7) giving 0.462 g (52%) of the desired compound (**7**) as a yellowish oil: GC-MS (70 eV) *m*/*z* (%): 343 (M^+^ < 1), 151 (100). The corresponding hydrobromide (**7**^.^HBr) was obtained dissolving the free base in 2 mL of 2 M HBr and azeotropically removing water (toluene/abs EtOH). The oil obtained was crystallized from abs EtOH/*i*Pr_2_O giving 0.40 g (36%) of white crystals: mp 126–127 °C; ^1^H NMR (CDCl_3_, 300 MHz): δ 1.49 (t, *J* = 7.3 Hz, 3H, C*H*_3_CH_2_), 2.95–3.12 (m, 2H, CH_3_C*H*_2_), 3.15–3.28 (m, 4H, C*H*_2_C*H*_2_), 3.88 (s, 6H, 2 × C*H*_3_O), 4.33 (d, *J* = 5.3 Hz, 2H, C*H*_2_N), 5.91 (s, 2H, OC*H*_2_O), 6.62–6.72 (m, 3H, phenyl *H*C-4 + benzodioxole *H*C-4 + benzodioxole *H*C-7), 7.00 (dd, *J* = 8.2, 1.2 Hz, 1H, benzodioxole *H*C-6), 7.15 (apparent t, 1H, phenyl *H*C-5), 7.43 (dd, *J* = 7.9, 1.5 Hz, 1H, phenyl *H*C-6), 11.2 (br s, 1H, NH^+^); ^13^C NMR (CDCl_3_, 300 MHz): δ 9.1 (1C, *C*H_3_CH_2_), 30.0 (1C, *C*H_2_), 47.4 (1C, *C*H_2_), 50.0 (1C, *C*H_2_), 53.0 (1C, *C*H_2_), 55.8 (1C, *C*H_3_O), 61.3 (1C, *C*H_3_O), 101.0 (1C, O*C*H_2_O), 108.6 (1C, Ar-*C*H), 109.0 (1C, Ar-*C*H), 114.4 (1C, Ar-*C*H), 121.6 (1C, *C*q), 121.9 (1C, Ar-*C*H), 124.5 (1C, Ar-*C*H), 125.0 (1C, Ar-*C*H), 129.7 (1C, *C*q), 146.7 (1C, benzodioxole *C*-7a or 3a), 148.0 (1C, benzodioxole *C*-3a or 7a), 148.4 (1C, phenyl *C*-2 or *C*-3), 152.6 (1C, phenyl *C*-3 or *C*-2). Anal. Calcd for C_20_H_25_NO_4_^.^HBr: C, 56.61; H, 6.18; N, 3.30. Found: C, 56.85; H, 6.27; N, 3.52.

*N*-[(1,3-Benzodioxol-5-yl)ethyl]-*N*-(2,3-dimethoxybenzyl)butan-1-amine hydrobromide (**8**^.^HBr)

Prepared as described above for **7** starting from **5a** and 1-iodobutane in 71% yield. GC-MS (70 eV) *m*/*z* (%): 328 (M^+^-43 < 1), 151 (100).

Data for **8**^.^HBr (white crystals): mp 103–104 °C; ^1^H NMR (CDCl_3_, 300 MHz): δ 0.92 (t, *J* = 7.3 Hz, 3H, C*H*_3_CH_2_), 1.25–1.40 (m, 2H, CH_3_C*H*_2_), 1.82–1.98 (m, 2H, CH_3_CH_2_C*H*_2_), 2.90–3.20 (m, 6H, NC*H*_2_C*H*_2_ + CH_3_CH_2_CH_2_C*H*_2_), 3.88 (s, 6H, 2 x C*H*_3_O), 4.34 (s, 2H, C*H*_2_N), 5.91 (s, 2H, OC*H*_2_O), 6.62–6.74 (m, 3H, phenyl *H*C-4 + benzodioxole *H*C-4 + benzodioxole *H*C-6), 7.00 (d, *J* = 8.2 Hz, 1H, benzodioxole *H*C-7), 7.14 (apparent t, 1H, phenyl *H*C-5), 7.41 (dd, *J* = 7.6, 1.2 Hz, 1H, phenyl *H*C-6), 11.15 (br s, 1H, NH^+^); ^13^C NMR (CDCl_3_, 500 MHz): δ 13.5 (1C, *C*H_3_CH_2_), 20.2 (1C, *C*H_2_), 25.5 (1C, *C*H_2_), 30.1 (1C, *C*H_2_), 50.5 (1C, *C*H_2_), 52.4 (1C, *C*H_2_), 53.7 (1C, *C*H_2_), 55.8 (1C, *C*H_3_O), 61.3 (1C, *C*H_3_O), 101.0 (1C, O*C*H_2_O), 108.6 (1C, Ar-*C*H), 109.1 (1C, Ar-*C*H), 114.4 (1C), 121.9 (2C, Ar-*C*H), 124.5 (1C), 124.9 (1C, Ar-*C*H), 129.7 (1C, *C*q), 146.7 (1C, benzodioxole *C*-7a or 3a), 148.0 (1C, benzodioxole *C*-3a or 7a), 148.4 (1C, phenyl *C*-2 or *C*-3), 152.7 (1C, phenyl *C*-3 or *C*-2). Anal. Calcd for C_22_H_29_NO_4_^.^HBr^.^0.5H_2_O: C, 57.27; H, 6.77; N, 3.04. Found: C, 57.14; H, 6.69; N, 3.15.

*N*-[(1,3-Benzodioxol-5-yl)ethyl]-*N*-(2,3-dimethoxybenzyl)hexan-1-amine hydrobromide (**9**^.^HBr)

Prepared as described above for **7** starting from **5a** and 1-iodohexane in 80% yield. HRMS (QTOF, *m*/*z*) calcd for C_24_H_33_NO_4_: 400.2482 ([M + H] ^+^); found: 400.2491.

Data for **9**^.^HBr (white crystals): mp 116–117 °C; ^1^H NMR (DMSO-*d*_6_, 500 MHz): δ 0.85 (t, *J* = 6.8 Hz, 3H, C*H*_3_CH_2_), 1.20–1.30 (m, 6H, C*H*_2_hexyl), 1.69 (apparent br s, 2H, C*H*_2_hexyl), 2.90–2.98 (m, 2H, NC*H*_2_hexyl), 3.00–3.08 (m, 2H, C*H*_2_CH_2_N), 3.10–3.25 (m, 2H, CH_2_C*H*_2_N), 3.81 (s, 3H, C*H*_3_O), 3.83 (s, 3H, C*H*_3_O), 4.35 (d, *J* = 4.9 Hz, 2H, C*H*_2_N), 5.97 (s, 2H, OC*H*_2_O), 6.70 (dd, *J* = 8.1, 1.2 Hz, 1H, phenyl *H*C-4), 6.80–6.88 (m, 2H, benzodioxole *H*C-6 + benzodioxole *H*C-4), 7.12–7.20 (m, 3H, phenyl *H*C-6 + phenyl *H*C-5 + benzodioxole *H*C-7), 9.23 (br s, 1H, NH^+^); ^13^C NMR (DMSO-*d*_6_, 500 MHz): δ 14.3 (1C, *C*H_3_CH_2_), 22.3 (1C, *C*H_2_), 23.3 (1C, *C*H_2_), 26.1 (1C, *C*H_2_), 29.2 (1C, *C*H_2_), 31.1 (1C, *C*H_2_), 51.3 (1C, *C*H_2_), 52.9 (1C, *C*H_2_), 54.0 (1C, *C*H_2_), 56.3 (1C, *C*H_3_O), 61.2 (1C, *C*H_3_O), 101.4 (1C, O*C*H_2_O), 108.8 (1C, Ar-*C*H), 109.6 (1C, Ar-*C*H), 115.2 (1C, Ar-*C*H), 122.3 (1C, Ar-*C*H), 124.0 (1C, *C*q), 124.2 (1C, Ar-*C*H), 124.8 (1C, Ar-*C*H), 130.9 (1C, *C*q), 146.5 (1C, benzodioxole *C*-7a or 3a), 147.8 (1C, benzodioxole *C*-3a or 7a), 148.2 (1C, phenyl *C*-2 or *C*-3), 152.8 (1C, phenyl *C*-3 or *C*-2). Anal. Calcd for C_24_H_33_NO_4_^.^0.9HBr: C, 61.48; H, 7.28; N, 2.99. Found: C, 61.20; H 7.15; N, 3.33.

### 2.2. Antibacterial Assay

The antibacterial activity of berberine chloride was tested against some bacterial strains.

We consider in this study, Gram-positive and Gram-negative bacteria. Antibacterial activity was assessed against strains belonging to the ATCC collection as *Bacillus subtilis ATCC 6633, Staphylococcus aureus ATCC 29213, Enterococcus faecalis ATCC 29212, Escherichia coli ATCC 25922, Klebsiella pneumoniae ATCC 13883.*

We used modified MIC determinations, according to the Clinical Laboratory Standards Institute (CLSI) guidelines. MIC values are given in µg/mL and were compared to MIC values for the standard antibacterial drug norfloxacin.

The bacterial species were cultured on Mueller Hinton agar (MHA, Oxoid) and each bacterial suspension was composed of 2–3 colonies of each strain taken from an MHA plate and dissolved in 2 mL of MHB (Mueller Hinton Broth). The resulting suspensions were diluted with 0.85% NaCl solution and then adjusted to 1 × 10^8^ CFU/mL (0.5 Mc Farland). The MICs of berberine chloride and norfloxacin were determined by the broth microdilution method, according to CLSI (Clinical and Laboratory Standard Institute Protocol M7A6 guidelines with some modifications to CLSI Protocol M7A6. The modifications regarded the dilutions of the product and some aspects that considered the control of sterility of microtiter wells and growing media during the evaluation of Minimal Inibhitory Concentration (MIC). The percentage of DMSO present in each well was controlled using a serial dilution of this solvent to establish the tolerance of each strain to DMSO. Consequently, the opportune percentage of solvent was used to determine the compound that had no adverse effect against the strains.

MICS (MICs, µg/mL) were established by the broth microdilution, using 96-well plates, as indicate by protocol guidelines (CLSI M7A8). Stock solution of the tested compound was obtained in DMSO. Then two-fold serial dilutions were plated in the suitable test medium and ranged between 1048 and 0.5 µg/mL. Precultures of each bacterial strain were prepared in Mueller–Hinton broth (MHB) and incubated at 37 °C until the growth ceased. The turbidity of bacterial cell suspension was measured at a wavelength of 625 nm using spectrophotometric method (Thermo Spectronic, Genesis 20); it should be 0.08 to 0.10 for the 0.5 McFarland standard, corresponding approximately to 10^8^ CFU (Colony Forming Units/mL). Then, the standardized suspension was diluted 1:100 with MHB to have 1–2 × 10^6^ CFU/mL. All wells were seeded with 100 µL of inoculum. A number of wells containing only inoculated broth as control growth were prepared. The plates were incubated at 37 °C for 24 h, and the MIC values were recorded as the last well containing no bacterial growth. The MIC was determined by using an antibacterial assayed repeated in duplicate. Norfloxacin was used as reference drug. MIC, expressed in μg/mL, was defined as the lowest concentration that did not result in any visible growth of the bacterial strains compared to their growth in the control well.

### 2.3. Molecular Docking Analysis

The ability of FtsZ to bind the berberine analogues was evaluated by docking analyses. The FtsZ protein was modeled using the I-TASSER Server. Berberine and compounds **5a**, **5b**, **6**–**9** were obtained using the LigParGen Server exploiting the Isomeric SMILES Code.

The protein-ligand model was constructed using the PatchDock Server [35] and the structures were then refined with the FireDock Server [36]. The Protein–Ligand Interaction Profiler (PLIP) Server [37] was used to define the interactions between the protein and the ligand. Finally, the Gibbs free energy (ΔG) values were predicted using the PRODIGY webserver. All the figures were generated through UCSF CHIMERA software.

### 2.4. Pharmacokinetic Parameters Prediction

Pharmacokinetic parameters were predicted in silico to get insights on drug-likeness properties of berberine and its analogues. The login-free website http://www.swissadme.ch/ (21 April 2021) [38] was used.

## 3. Results and Discussion

**Scheme 1:**
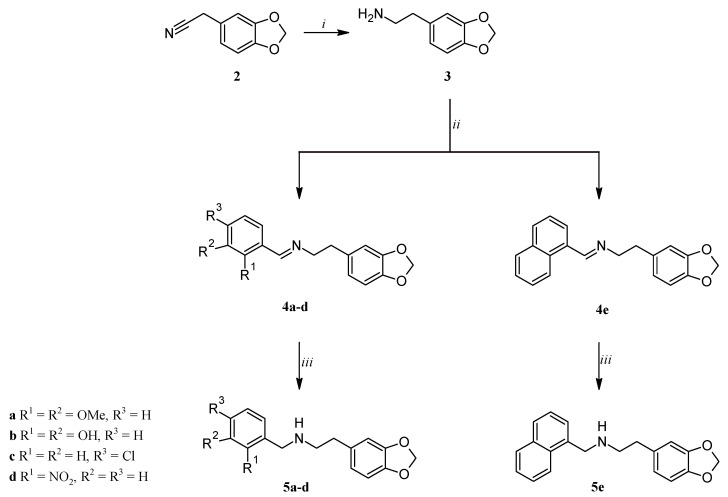
Reagents and conditions: (**i**) BMS, dry THF, 1 h, 0 °C, then 16 h, reflux; (**ii**) suitable benzaldehyde, 3 Å molecular sieves, dry CH_2_Cl_2_, overnight, room temp; (**iii**) NaBH_4_, MeOH/CH_2_Cl_2_, 2 h, reflux.

**Scheme 2:**
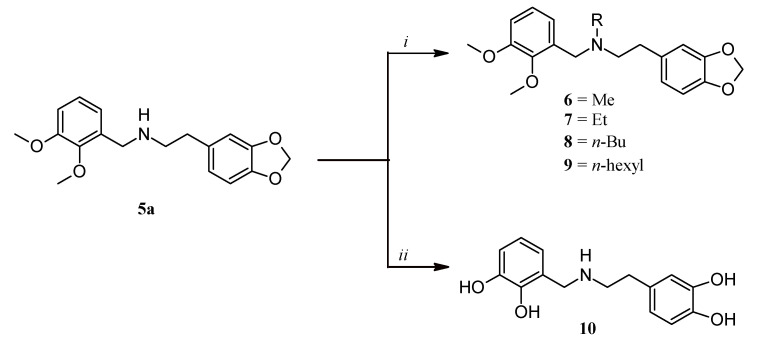
Reagents and conditions: (**i**) suitable 1-iodoalkane, K_2_CO_3_, abs EtOH, 24 h, room temp for compound **6**, reflux for compounds **7**–**9**; (**ii**) BBr_3_, dry CH_2_Cl_2_, 1 h, 0 °C, then 1 h, room temp.

**Scheme 3:**
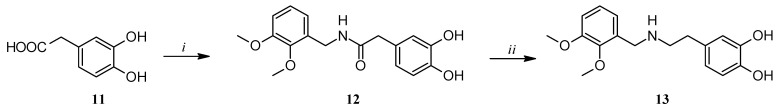
Reagents and conditions: (**i**) 2,3-dimethoxybenzylamine, EEDQ, Et_3_N, CHCl_3_, MW, 100 °C, 15 min; (**ii**) BMS, dry THF, 17 h, reflux.

We started from the observation that many of the antibacterial berberine derivatives reported in the literature (Figure 1) show flat and lipophilic structures unsuitable for oral administration and that some relevant structural alerts such as the quaternary pyridinium salt (structures A and B in Figure 1) and polycyclic aromatic hydrocarbons (structures B in Figure 1) are contained in their structures. Therefore, with the aim of identifying a novel series of berberine analogues possibly easier to synthesize and orally administrable according to the Lipinski’s rules of 5, the well-known structural simplification strategy was applied to berberine. A series of less flat analogues endowed with greater flexibility (**5a–e**, **Scheme 1**; **6**–**10**, Scheme 2; **13**, Scheme 3) were prepared and screened. In particular, compounds with unmasked hydroxyl groups (**5b**, **10**, **13**) were designed with the aim of improving the poor berberine water solubility. On the other hand, the idea of removing the methyl groups and/or the methylene bridge on the four oxygen atoms arose from the awareness that aromatic hydroxyl groups destabilize the cytoplasmic membrane leading to bacterial cell death [39,40]. Therefore, increased antibacterial activity was expected for the hydroxylated analogues of berberine. Finally, compounds **14** and **15**, previously obtained in our laboratory, were also evaluated as antibacterial agents.

### 3.1. Chemistry

Compounds **5a**–**e** were prepared as depicted in Scheme 1 [32]. 3,4-Methylendioxyphenylacetonitrile (**2**) was reduced with borane-methyl sulfide complex (BMS) and the so-obtained 2-(3,4-methylenedioxyphenyl)ethylamine (**3**) was reacted with the suitable benzaldehyde affording the corresponding imines **4a–e** which were in turn reduced with NaBH_4_ to give compounds **5a–e**.

*N*-alkylation of **5a** was carried out modifying a literature procedure [41] and afforded the tertiary amines **6**–**9** which were converted into their corresponding hydrobromide salts (**6**–**9**^.^HBr) by treating the free amines with aqueous HBr and azeotropically removing water (Scheme 2). Finally, the overall deprotection of **5a** with BBr_3_ gave compound **10** as the hydrobromide salt (**10**^.^HBr).

Compounds **5c**–**e** and **6**–**9** are novel. Compound **13** was obtained as reported in the literature [32] starting from 3,4-dihydroxyphenylacetic acid (**11**) which was condensed with 2,3-dimethoxybenzylamine, in the presence of 2-ethoxy-1-ethoxycarbonyl-1,2-dihydroquinoline (EEDQ) (Scheme 3) [42]. The desired compound **13** was finally obtained by reducing the amide **12** with BMS.

**Scheme 4:**
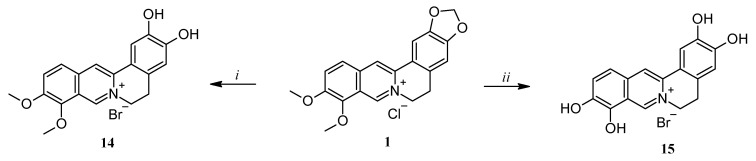
Reagents and conditions: (**i**) BBr_3_, dry CH_2_Cl_2_, 1 h, 0 °C, then 1 h, room temp; (**ii**) BBr_3_, dry CH_2_Cl_2_, 1 h, 0 °C, then 20 h, reflux.

Berberine derivatives **14** and **15** (Scheme 4) were prepared, as previously reported [32], by treating commercially available berberine chloride (**1**) with BBr_3_ in dry CH_2_Cl_2_.

### 3.2. Antibacterial Activity

The in vitro ability of all the synthesized compounds to inhibit the growth of selected pathogens belonging to the ATCC collection was evaluated through the microdilution broth method. In particular, the antibacterial activity was tested against three Gram-positive bacterial strains (*Bacillus subtilis ATCC 6633, Staphylococcus aureus* *ATCC 29213*, *Enterococcus faecalis ATCC 29212)* and two Gram-negative bacterial strains (*Escherichia coli ATCC 25922, Klebsiella pneumoniae ATCC 13883).* The results expressed as the Minimum Inhibitory Concentration (MIC, μg/mL), the lowest concentration required to inhibit the visible growth of microorganisms, are listed in Table 1.

As shown in Table 1, the removal of the methylene bridge of berberine greatly improved the antimicrobial activity against *S. aureus* and *K. pneumoniae*, with the MIC value of **14** being 8-fold lower than that of berberine on both strains. Conversely, the weak activity observed for 2,3,9,10-tetrahydroxyberberine bromide (**15**) against all tested strains might be assigned to both its poor solubility in the culture medium and the fast degradation that occurred within 24 h from solubilization. In agreement with observations on berberine (**1**) and its dealkylated derivative **14**, the berberine simplified analogue **5a** showed no activity at the highest tested doses thus confirming that the absence of hydroxyl groups is detrimental to the antibacterial activity, regardless of the acquired conformational flexibility. Interestingly, the presence of a catechol moiety distal from the nitrogen atom (compare **5a** with **13**) led to a remarkable increase of antibacterial activity, with the lowest MIC value being observed against *S. aureus* and *K*. *pneumoniae* (23.75 μg/mL). Since a similar behavior has also been observed for berberine and its demethylene derivative **14** on the same pathogens, these results suggest a correlation between the presence of two distal hydroxyl groups and the antibacterial activity against *S. aureus* and *K*. *pneumoniae*. With the same pattern of substituents, no activity was observed when the nitrogen atom was involved in an amide bond (**12**), thus highlighting the need for the presence of a basic nitrogen atom. Furthermore, based on the results obtained with compound **5b**, we speculated that the presence of a *N*-(2,3-dihydroxybenzyl) moiety provides a broad spectrum of antibacterial activity, although at concentrations higher than those of the most potent analogues of the series but still lower than that of berberine (128–256 μg/mL). The effects of both structural modifications, i.e., the presence of a distal and proximal catechol moiety with respect to the nitrogen atom, have been confirmed by the tetrahydroxy analog **10** that showed not only a broad spectrum of activity against all tested bacterial strains (as observed for compound **5b**) but also the lowest MIC values against *S. aureus* and *K*. *pneumoniae* (paralleling what observed for compound **13**). With the aim of replacing the electron-donating methoxy groups on the benzyl moiety with electron-withdrawing substituents (Cl, NO_2_), compounds **5c,d** were synthesized. Besides, the lipophilicity effect was also evaluated through the naphthyl analogue **5e**. Unlike berberine (**1**) and its simplified analogue **5a** whose MIC value was >512 μg/mL against all tested strains, all three new compounds showed a broad spectrum of activity, with the best results obtained for compound **5e** against *S. aureus*. Based on the results obtained so far, the only completely inactive compound within the secondary amine series **5a–e** was **5a** which maintains the same substituent pattern as berberine on the two aryl moieties but not on the amino function. With the aim of investigating the effect of the highest degree of nitrogen atom substitution so as to obtain compounds better resembling the berberine scaffold, a series of tertiary amines with increasing *N*-alkyl chains were prepared and tested (compounds **6**–**9**). Out of four *N*-alkyl derivatives (**6**–**9**), only the methyl analogue **6** displayed an interesting activity on the bacterial test species, with the lowest MIC value being observed against the Gram-negative *E. coli.* At the highest tested concentrations, all other tertiary amines (**7**–**9**) were found to be inactive on *E. coli*, as well as on all other bacterial strains. This result prompted us to hypothesize an optimal interaction between the smaller methyl substituent of compound **6** and the putative binding pocket of a bacterial target protein.

### 3.3. Molecular Docking Analysis

Since the inhibitory activity of berberine against FtsZ was previously reported [21,33,34

**Figure 3:**
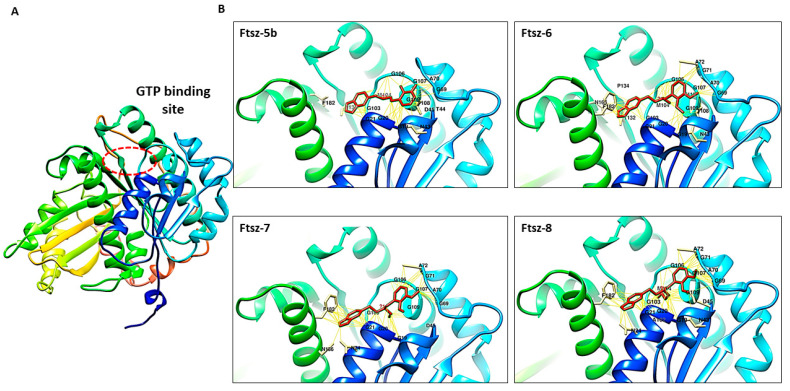

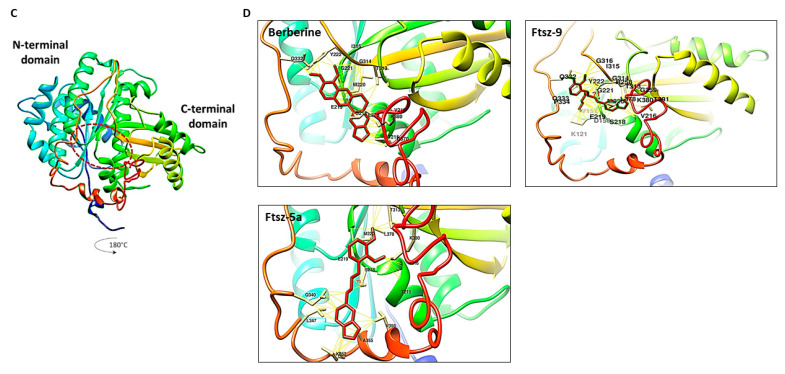
(**A**) FtsZ model obtained with I-TASSER Server; (**B**) predicted interactions between FtsZ and compounds **5b**, **6**–**8**; (**C**) FtsZ rotation of 180°; (**D**) predicted interactions with berberine and compounds **5a**, **9**. The interactions prediction was obtained using the Protein-Ligand Interaction Profiler (PLIP) Server. The residues involved in the interaction and the connection between the residues are in yellow. Images were generated by the CHIMERA software.

], modeling studies were carried out to verify whether some of the synthesized compounds were able to interact with this protein. FtsZ is a bacterial tubulin homolog responsible for the Z-ring formation during cell division. Several inhibitors, including both synthetic and natural products, have been tested as promising antibacterial agents by impairing cell division inducing bacterial cell death [43,44,45,46,47,48,49,50]. Biochemical and genetic evidence for berberine inhibition of FtsZ have also been reported [21,33,34]. According to the peculiar behaviour of the tertiary amine **6** on *E. coli*, compared to the inactive homologues **7**–**9** (Table 1), the possible interaction between the *N*-alkylated analogues **6**–**9** and the *E. coli* FtsZ protein was investigated in silico by docking simulation experiments using PatchDock Server and FireDock Server. The *E. coli* FtsZ was modeled with the I-TASSER Webserver whilst the LigParGen Server was used to obtain the structures of berberine and its analogues under evaluation. Starting from the tertiary amine **6** showing the lowest MIC value (128 μg/mL), the whole series of the tertiary amines **6**–**9** together with the corresponding *N*-dealkylated analogue **5a** were investigated. Moreover, the secondary amine **5b** was also evaluated since it was twice as potent as **5a** on *E. coli* (MIC values 256 μg/mL and >512 μg/mL, respectively). Therefore, possible different interactions of the catechol moiety of **5b** with respect to the corresponding methoxy groups of **5a** with the target protein were investigated. According to docking calculations, compounds **5b** and **6**–**8** may bind the protein active site within the GTP-binding pocket (Figure 3A,B), thus suggesting a possible competitive inhibition mechanism on the GTPase activity of the protein. Furthermore, a detailed analysis of the interactions between these berberine analogues and the protein showed that several key residues for the enzymatic activity of FtsZ, including Gly20–22, Gly72, Ala73, Gly107–109, and Thr133 [51], might be involved in the binding.

**Figure 4:**
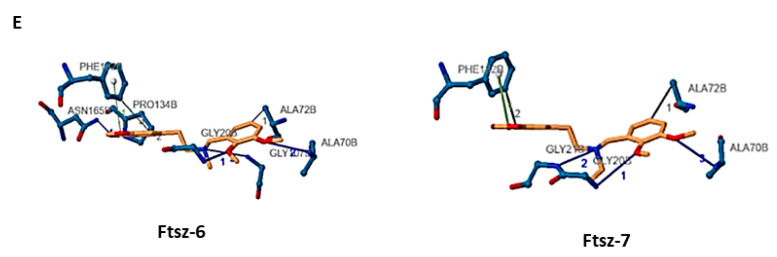
π-Stacking interaction of the benzodioxole ring of compounds **6** and **7** with Phe182 (see the text for details).

It is worth noting that, besides the hydrophobic interactions and hydrogen bonds identified for compounds **5b** and **6**–**8**, only for the *N*-methyl (**6**) and *N*-ethyl (**7**) derivatives an additional π-stacking interaction with Phe182 was predicted. A more in-depth analysis of the obtained data (Figure 4) showed that only the benzodioxole moiety of compound **6** exhibits a favored T-shaped conformation with Phe182, with a dihedral angle of about 90°, whilst the corresponding moiety in its analogue **7** presented a less favorable 70° solid angle in the interaction with Phe182. Therefore, the *N*-methyl derivative **6** is the analogue originating the strongest interactions with the protein, in agreement with the results obtained in the MIC assays against *E. coli*.

Surprisingly, the homologues **5a** and **9** showed a completely different behavior from the other members of the series. Analogously to berberine, these compounds interact with FtsZ within a different binding site near the C terminus of the protein (Figure 3C,D). Conceivably, it may be assumed that the GTP-binding site can only host short alkyl substituents whereas the relatively bulky *N*-hexyl chain of compound **9** might be unable to enter this small pocket. On the other hand, the difficult fitting of substituents bulkier than the ethyl group was already observed for compound **8** whose butyl chain is forced in a folded conformation to interact with this pocket (Figure 3B). Based on these results, only the *N*-methyl and *N*-ethyl short substituents (analogues **6** and **7**) allow the molecule to arrange in the FtsZ active site in a suitable conformation for a favorable interaction. As the size of the alkyl increases the interaction with the GTP binding site worsens, finally being totally prevented in the presence of the relatively bulkier hexyl chain. The secondary amine **5a** also binds FtsZ near the C terminus of the protein in the same way as berberine and compound **9** very likely because it cannot originate any interaction with the lipophilic pocket being devoid of nitrogen substituents. Finally, the demethylated analogue **5b** takes advantage of the presence of two hydrogen bond donor hydroxyl groups thus being capable of interacting with the GTP-binding pocket despite the lack of nitrogen substituents like **5a**.

As reported in Table 2, the Gibbs free energy analysis performed using the PRODIGY webserver revealed that berberine and the compounds **5a**, **5b**, **6**–**9** showed negative ΔG values suggesting the formation of protein–ligand complexes with comparable stability. Among the series of homologous compounds interacting with FtsZ (**5a**, **5b**, **6**–**9**), compound **6** originated the most stable complex with a predicted ΔG value of –7.96 kcal/mol (very similar to the result obtained for berberine) in agreement with both the above considerations and the MIC assay results. On the contrary, the 2,3-hydroxybenzyl analogue **5b** showed the highest ΔG value (−7.45 kcal/mol), thus demonstrating the usefulness of the methyl groups masking oxygen atoms for a strongest interaction with FtsZ as in the corresponding analogue **5a**. However, this result seems to be in contrast with the lower MIC value obtained for compound **5b** compared to the analogue **5a**, thus possibly suggesting a different mechanism of action, independent of FtsZ inhibition, for those compounds bearing a catechol moiety.

### 3.4. Pharmacokinetic Parameters Prediction

Some relevant pharmacokinetic parameters and likenesses of the four compounds most strictly related to berberine (**5b**, **6**, **10**, and **13**) were further investigated in silico using the SwissADME online platform to predict their possible oral administration. The calculated physicochemical properties are reported in Table 3 where berberine shows the highest lipophilicity as well as the lowest water solubility and flexibility (number of rotatable bonds), as expected. Berberine and compound **10** seemed to be unable to cross the blood–brain barrier (BBB), thus presumably being not toxic at the central nervous system. Although all the compounds were drug-like since no violations to Lipinski’s rules of 5 have been provided, only berberine showed violations to the lead-likeness indicators, with its molecular weight (MW) being > 350 and XLOGP > 3.5. These values suggest that the insertion of further substituents on berberine structure is not advisable, mostly when orally administered drugs are to be obtained. On the contrary, our low-molecular-weight analogues may be considered as berberine fragments and, according to the fragment-based drug discovery approach, structural modifications can be gradually introduced to further increase their potency, which is already higher than their parent compound. Overall, this study indicates that our simplified models of berberine are more suitable starting points than berberine itself for further drug development.

## 4. Conclusions

A series of non-quaternized analogues of berberine endowed with higher structural flexibility and water solubility were prepared according to the well-known structural simplification strategy and tested as antibacterial agents. Besides being devoid of some relevant structural alerts such as a quaternary pyridinium nitrogen and polycyclic aromatic hydrocarbon core, our most potent compounds have higher ligand efficiency (LE) indexes than berberine and its previously reported derivatives. While being less potent than the latter, the simplified berberine analogues reported herein are attractive since it is now widely accepted that low-affinity hit compounds should be prioritized for further optimization provided they have relatively high ligand efficiency. In particular, the best results were obtained when hydroxyl groups of berberine and its analogues have been unmasked. The presence of a catechol moiety in a distal position to the nitrogen atom or the replacement of the 2,3-dimethoxyphenyl ring with a napthyl moiety led to the highest antibacterial activity observed, in particular against *S. aureus* and *K. pneumoniae*, whilst the presence of an *N*-(2,3-dihydroxybenzyl) moiety led to the widening of the antibacterial activity against all the Gram-positive and Gram-negative bacteria tested. Moreover, interesting activity on *E. coli* was observed for the tertiary amine bearing a methyl group on the nitrogen atom and, according to the results of molecular modeling studies, this activity might be related to the inhibition of FtsZ.

Overall, our study demonstrates that structural simplification of natural compounds is a powerful strategy to easily synthesize small molecules more potent than their parent compounds and, being structural fragments with low MW, further functionalizable so as to obtain compounds still retaining the Lipinski requirement for oral administration. Starting from the observation that 8- and 9-substituted berberine analogues show much greater potency than their parent compound [52], similar substitutions on the corresponding positions of our simplified analogues will be further explored.

## Figures and Tables

**Table 1:** Antibacterial activity (MIC, μg/mL) of berberine (**1**), berberine analogues (**5a–e**, **6**–**10**, **12**, **13**), and berberine derivatives (**14**, **15**).

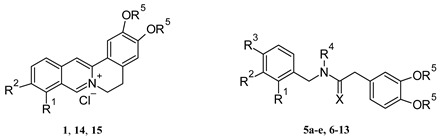											
**Compd**	**R^1^**	**R^2^**	**R^3^**	**R^4^**	**R^5^**	**X**	**Gram-Positive**			**Gram-Negative**	
							*B. subtilis* ATCC 6633	*S. aureus* ATCC 29213	*E. faecalis* ATCC 29212	*E. coli* ATCC 25922	*K. pneumoniae* ATCC 13883
**1**	OCH_3_	OCH_3_	–	–	–CH_2_–	–	>512	512	>512	512	>512
**5b^.^HCl**	OH	OH	H	H	–CH_2_–	H_2_	128	128	256	256	128
**5c^.^HCl**	H	H	Cl	H	–CH_2_–	H_2_	n.d.	64	128	128	128
**5d^.^HCl**	NO_2_	H	H	H	–CH_2_–	H_2_	n.d.	128	128	256	512
**5e^.^HCl**	CH=CHCH=CH		H	H	–CH_2_–	H_2_	n.d.	32	64	128	128
**6^.^HBr**	OCH_3_	OCH_3_	H	CH_3_	–CH_2_–	H_2_	256	512	512	128	256
**10**	OH	OH	H	H	H	H_2_	256	64	256	256	64
**13**	OCH_3_	OCH_3_	H	H	H	H_2_	>512	23.75	>512	>512	23.75
**14**	OCH_3_	OCH_3_	–	–	H	–	>512	64	512	256	64

MIC values of compounds **5a**, **7**–**9**, **12**, and **15** were ≥512 μg/mL; n.d. not determined.

**Table 2:** Interactions between FtsZ protein and berberine or its analogues.

Protein-Ligand	Interaction				Gibbs Free Energy Variation(ΔG, kcal/mol)
	*Hydrophobic interactions*	*Hydrogen bonds*	*Noncovalent interaction*	*π-Stacking*	
**FtsZ-berberine**	2 Met220, Leu378	2 Arg258, Lys380	-	-	−8.02
**FtsZ-5b**	1 Asp45	7 Ala70, Ala72, Gly107, Thr108, Gly109, Asn165	1 Phe182	-	–7.45
**FtsZ-5a**	5 Glu219, Gln340, Leu347, Val358, Leu378	2 Thr313, Lys 380	-	-	–7.87
**FtsZ-6**	3 Ala72, Pro134, Phe182	4 Gly20, Ala70, Gly107, Asn165	-	1 Phe 182	–7.96
**FtsZ-7**	2 Ala72, Phe 182	3 Gly20, Gly21, Ala71	-	1 Phe182	–7.86
**FtsZ-8**	2 Ala72, Phe182	3 Gly21, Gly21, Thr108	-	-	–7.85
**FtsZ-9**	6 Val157, Glu219, Met220, Tyr222, Pro334, Leu278	4 Arg258, Arg258, Thr313, Gln332	1 Lys380	-	–7.71

**Table 3:** Summary of calculated pharmacokinetic properties of berberine and the most strictly related compounds (**5b**, **6**, **10**, and **13**) by SwissADME ^a^.

Compd.	MW	*c*log *P*^b^	Water Solubility	BBB Permeability	Drug-Likeness ^c^	Lead-Likeness ^d^
Berberine	371	4.42	Poorly soluble	No	Yes	No ^e^
**5b**	287	2.90	Moderately soluble/soluble	Yes	Yes	Yes
**6**	329	3.47	Moderately soluble/soluble	Yes	Yes	Yes
**10**	275	2.38	Soluble	No	Yes	Yes
**13**	303	2.48	Moderately soluble/soluble	Yes	Yes	Yes

^a^ login-free website http://www.swissadme.ch/ (21 April 2021); ^b^ XLOGP3: atomistic and knowledge-based method calculated by XLOGP program, version 3.2.2, courtesy of CCBG, Shanghai Institute of Organic Chemistry; ^c^ all the compounds showed no violation to the Lipinski’s rules of 5; ^d^ Implemented from Teague SJ. 1999 Angew. Chem. Int. Ed.: 250 ≤ MW ≤ 350, XLOGP ≤ 3.5, rotatable bonds ≤ 7; ^e^ 2 violations: MW > 350 and XLOGP > 3.5.

## Data Availability

No data are available.
